# Feasibility of indocyanine green (ICG) fluorescence in *ex vivo* pathological dissection of colorectal lymph nodes—a pilot study

**DOI:** 10.3389/pore.2024.1611853

**Published:** 2024-08-29

**Authors:** Lorand Lakatos, Ildiko Illyes, Andras Budai, Viktoria Bencze, Attila Szijarto, Andras Kiss, Balazs Banky

**Affiliations:** ^1^ Department of Surgery, Transplantation and Gastroenterology, Semmelweis University, Budapest, Hungary; ^2^ Department of Pathology, Forensic and Insurance Medicine, Semmelweis University, Budapest, Hungary

**Keywords:** indocyanine green, colorectal cancer, lymph node, *ex vivo*, colorectal resection

## Abstract

Accurate lymph node (LN) retrieval during colorectal carcinoma resection is pivotal for precise N-staging and the determination of adjuvant therapy. Current guidelines recommend the examination of at least 12 mesocolic or mesorectal lymph nodes for accurate staging. Traditional histological processing techniques, reliant on visual inspection and palpation, are time-consuming and heavily dependent on the examiner’s expertise and availability. Various methods have been documented to enhance LN retrieval from colorectal specimens, including intra-arterial *ex vivo* methylene blue injection. Recent studies have explored the utility of indocyanine green (ICG) fluorescence imaging for visualizing pericolic lymph nodes and identifying sentinel lymph nodes in colorectal malignancies. This study included 10 patients who underwent colon resection for malignant tumors. During surgery, intravenous ICG dye and an endoscopic camera were employed to assess intestinal perfusion. Post-resection, *ex vivo* intra-arterial administration of ICG dye was performed on the specimens, followed by routine histological processing and an ICG-assisted lymph node dissection. The objective was to evaluate whether ICG imaging could identify additional lymph nodes compared to routine manual dissection and to assess the clinical relevance of these findings. For each patient, a minimum of 12 lymph nodes (median = 25.5, interquartile range = 12.25, maximum = 33) were examined. ICG imaging facilitated the detection of a median of three additional lymph nodes not identified during routine processing. Metastatic lymph nodes were found in four patients however no additional metastatic nodes were detected with ICG assistance. Our findings suggest that *ex vivo* intra-arterial administration of indocyanine green dye can augment lymph node dissection, particularly in cases where the number of lymph nodes retrieved is below the recommended threshold of 12.

## Introduction

Following the resection for colorectal carcinoma, the number of retrieved regional lymph nodes is important for determining the stage and adjuvant therapy. For accurate N-staging, as many as possible, but at least 12 mesocolic or mesorectal lymph nodes need to be histologically examined [1].

In the pathological processing of colorectal specimens, the traditional dissection “performed by visual inspection and palpation” is a time-consuming method. In addition, the effectiveness of lymph node harvesting significantly depends on the experience of the examiner and the time available. The technical difficulty is well illustrated by the fact that 80% of mesenteric lymph nodes are less than 3 mm in diameter, and 50% of metastatic lymph nodes are less than 5 mm in diameter [2].

Additionally, in rectal cancer cases, post-neoadjuvant total mesorectal excision (TME) dissection results in a typically low number of extracted lymph nodes, making even the threshold of 12 hard to achieve [3].

There are many methods known in the literature to facilitate the extraction of lymph nodes from a colorectal specimen, including intra-arterial *ex vivo* Methylene Blue specimen loading [4], fat-clearance techniques [5], and even the employment of extra pathology assistant staff [6].

*In vivo*, intraoperative fluorescence imaging of subserosal-injected indocyanine green (ICG) has recently been proven to be a promising method for imaging lymphatic vessels and lymph nodes [7]. Several studies have investigated peritumoral submucosal ICG injection by endoscopy to identify sentinel lymph nodes in colorectal cancer; however, the results are still controversial [8].

ICG, on the other hand, has been introduced as an effective tool to qualitatively and quantitatively verify adequate blood supply to the resection lines during colorectal resection [9]. Under near-infrared excitation (with the help of a laser light source and image processing suitable for fluorescence detection), intravenous injection of ICG solution helps to differentiate between well- and under-perfused tissues.

ICG has a relatively short half-life in the blood (approximately 2–4 min) and is quickly distributed to various tissues through systemic circulation. Combining the two phenomena that intra-arterial dyes accumulate in mesenteric lymph nodes (as in methylene blue studies) [10], and that ICG can be used for lymph node detection, we postulated that lymph node harvesting in histologic specimen processing may be enhanced by the ICG fluorescence technique. The aim of this study was to evaluate the feasibility and efficacy of the intraarterial ICG technique to facilitate lymph node dissection in colorectal resection.

## Materials and methods

**FIGURE 1:**
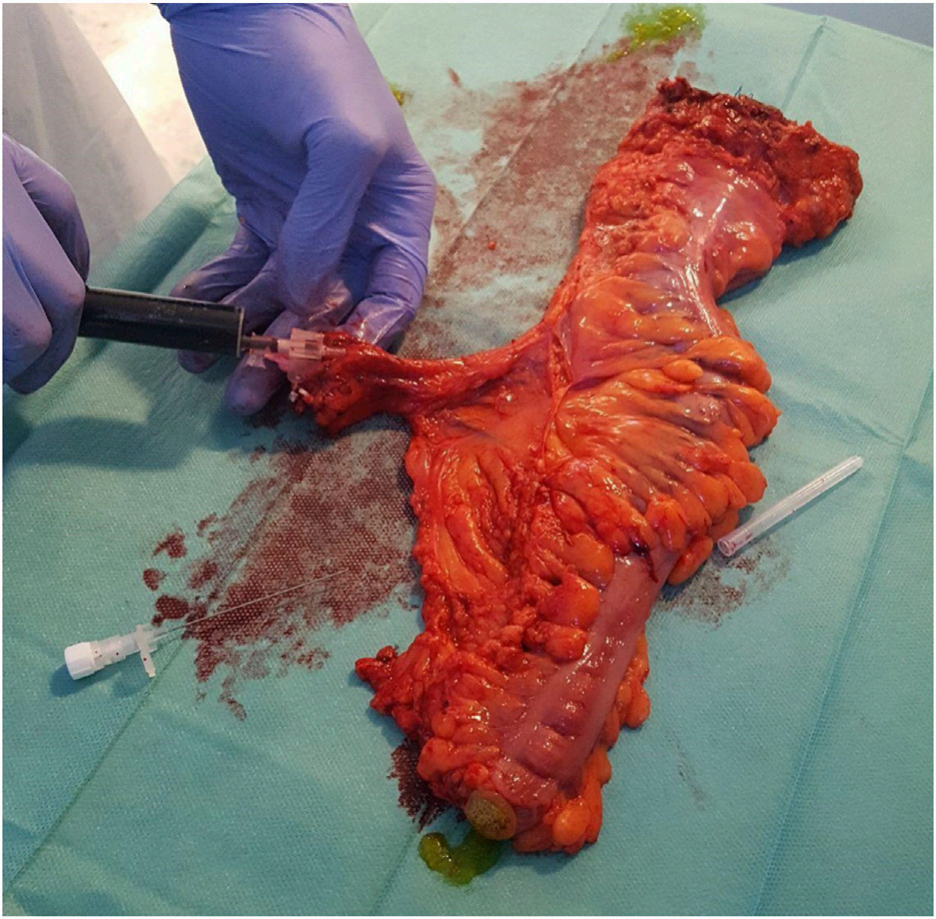
Cannulation of the supplying artery and ICG perfusion of the extracted specimen Ensure that all the figures, tables, and captions are correct, and that all figures are of the highest quality/resolution. You may upload improved figures to the Production Forum. If so, please describe in visual terms the exact changes(s) made to help us confirm that the updated version has been used in the finalized proof. Please note that figures and tables must be cited sequentially, per the author guidelines.

In our pilot study, we enrolled ten consecutive patients with colorectal cancer who underwent colorectal resection. We used the Medtronic Elevision™ IR platform imaging system with Verdye™ indocyanine dye as a fluorophore, to perform ICG fluorescence imaging (ICG-FI). The dye in powder form was dissolved in sterile distilled water for injection according to the manufacturer’s instructions, and a bolus intravenous injection of 0.05 mg/kg ICG dye was administered during surgery before colorectal resection as a routine perfusion check. After specimen extraction, arterial cannulation and a second shot of 0.05 mg/kg dose of direct intraarterial ICG were performed *ex vivo* under fluorescence imaging (Figure 1).

**FIGURE 2:**
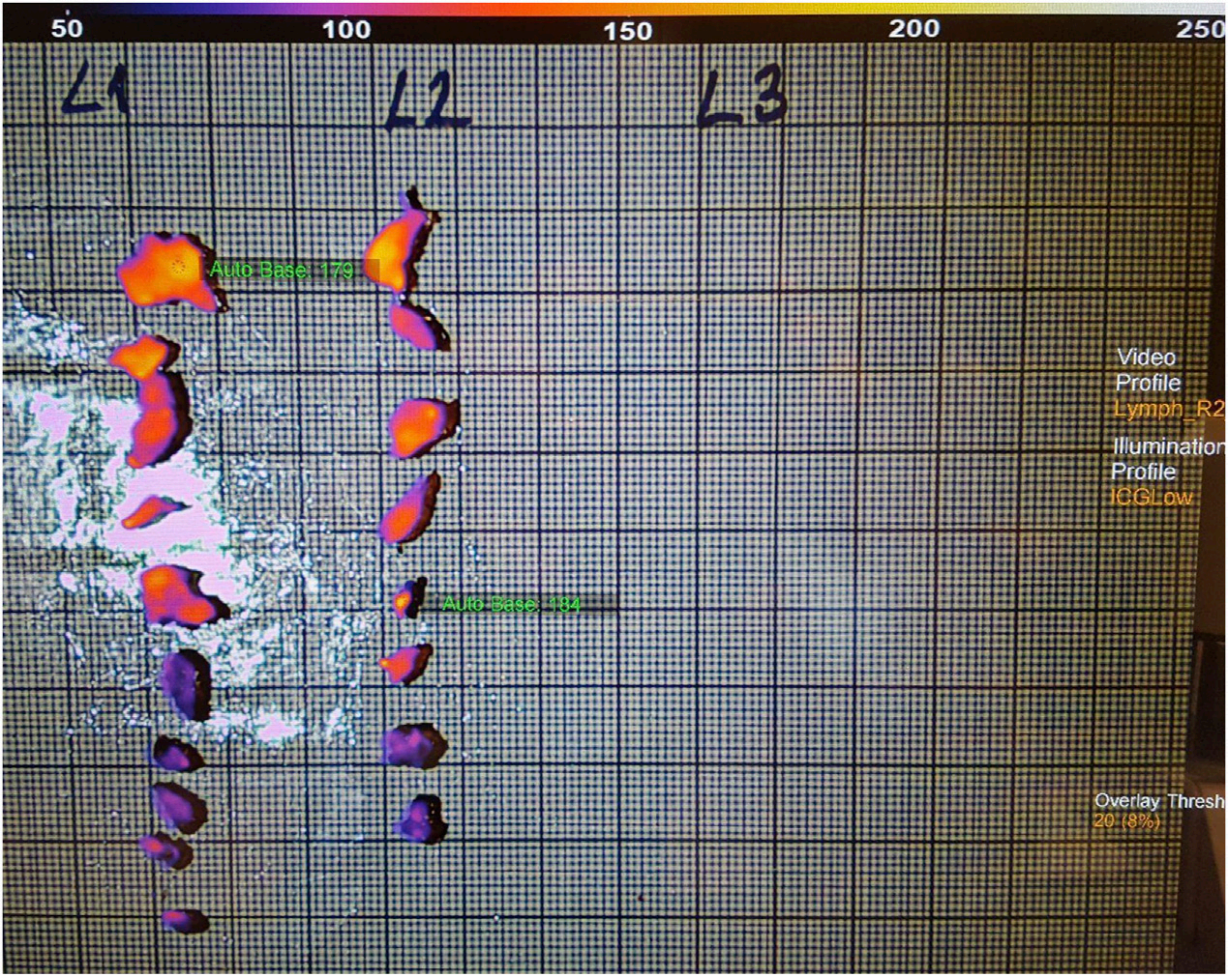
Fluorescence imaging of the extra harvested lymph nodes.

The specimen was then routinely processed after a 24-hour incubation in a 4% buffered formaldehyde solution and sent for traditional pathology grossing. After conventional lymph node dissection carried out by a pathologist under naked eye control and traditional palpation technique, each specimen was re-examined using ICG-FI. This was possible as formaldehyde does not compromise fluorescence excitability [11]. The additionally identified lymph nodes were sent for further pathological examination (Figure 2). The lymph node numbers of the primary (conventional) harvesting and the ICG-fluorescence-enhanced extra lymph node numbers were recorded. Lymph node sizes were measured as well during the pathological routine workup using a conventional light microscope.

**FIGURE 3:**
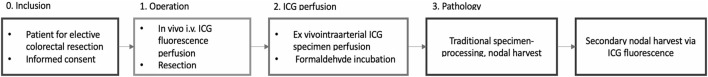
Study design.

A summary of the study design is shown in Figure 3.

In order to exclude or highlight the Hawthorne effect [12] we compared lymph node yields to a historical cohort of 30 consecutive patients with colorectal cancer with similar patient and clinical characteristics (propensity-matched cases).

### Statistical work-up

Demographic data, cancer characteristics, lymph node numbers and lymph node sizes were analyzed. The normality of the distributions of continuous variables was tested with the Shapiro-Wilk test. Comparisons were made using the Student’s paired sample T-test for normally distributed variables and the Mann-Whitney U-test for non-Gaussian distributed ones. Differences with *p* < 0.05 were considered significant. The propensity score was calculated by binal logarithmic regression, covariates were T and N stages, neoadjuvant therapy and type of resection. After propensity score matching we carried out the Mann-Whitney U test using mean ranks to determine whether there was a significant increase in lymph node yield with ICG assistance in addition to traditional harvesting. Statistical analysis was performed using SPSS (IBM^©^ SPSS^©^ statistics 27) software.

## Results

Patient characteristics and tumor characteristics are shown in Tables 1, 2.

**TABLE 1:** Patient characteristics.

Mean age	73
Sex ratio	1:1
Right hemicolectomy	3
Transverse colon resection	1
Rectal resection	6

**TABLE 2:** Tumor characteristics.

Patient #	T stage	N stage	Neoadjuvant therapy
1	3	0	Yes
2	4a	1b	No
3	3	1b	No
4	1	0	Yes
5	2	0	Yes
6	3	2a	No
7	2	0	No
8	2	0	Yes
9	4a	1a	No
10	4b	0	No

### Number of harvested lymph nodes

A median of 21.5 lymph nodes was identified in the experimental population by the initial conventional pathological workup which was supplemented by ICG fluorescence imaging to a median of 25.5 lymph nodes (Figure 6).

**FIGURE 4:**
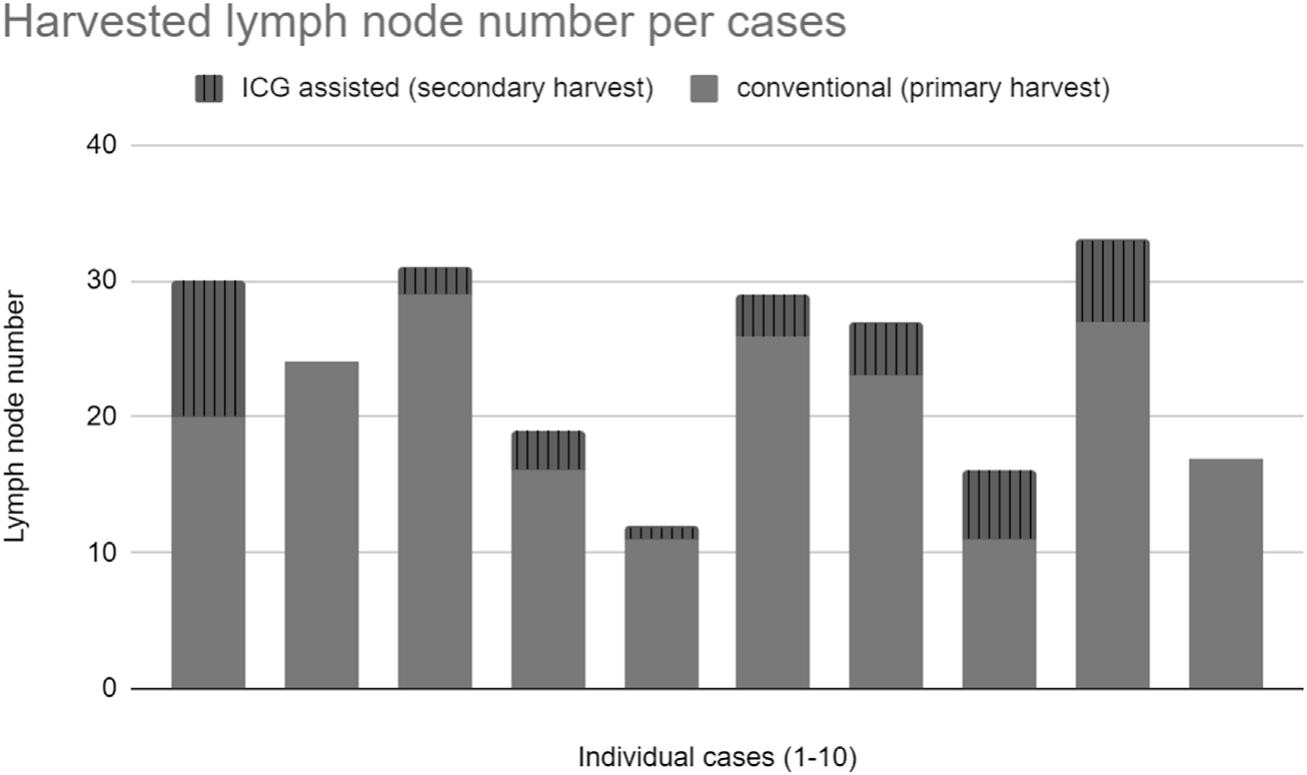
Total harvested lymph nodes.

ICG fluorescence imaging identified an additional 34 lymph nodes in 10 cases that were not detected by traditional pathological examination (Figure 4).

### Metastatic lymph nodes

A total of 11 metastatic lymph nodes were identified in 4 out of 10 specimens. No metastatic lymph node was identified by ICG.

### Size of harvested lymph nodes

**FIGURE 5:**
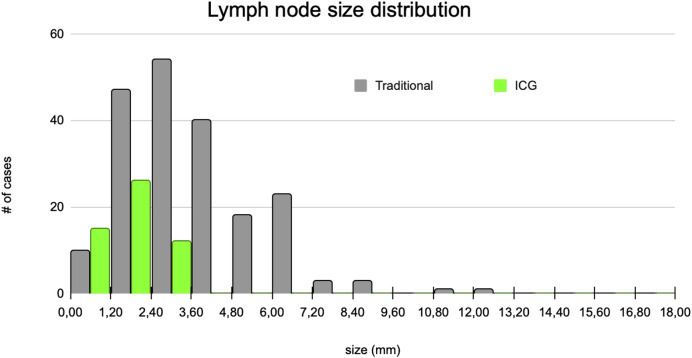
Lymph node size distributions.

Of all metastatic lymph nodes, 50% were smaller than 5 mm in diameter. The median size of lymph nodes harvested by the traditional pathological examination was 3 mm and 1.8 mm for lymph nodes identified with ICG (Figure 5).

The mean size of lymph nodes extracted by ICG assistance following traditional lymph node harvesting was half that of lymph nodes identified with conventional dissection (3.70 vs. 1.76 mm, SD 2.05 vs. 0.73 mm). We found that the smaller lymph nodes, which are more difficult to detect with traditional dissection methods, were more likely to be identified with ICG-FI.

### Historical cohort

**FIGURE 6:**
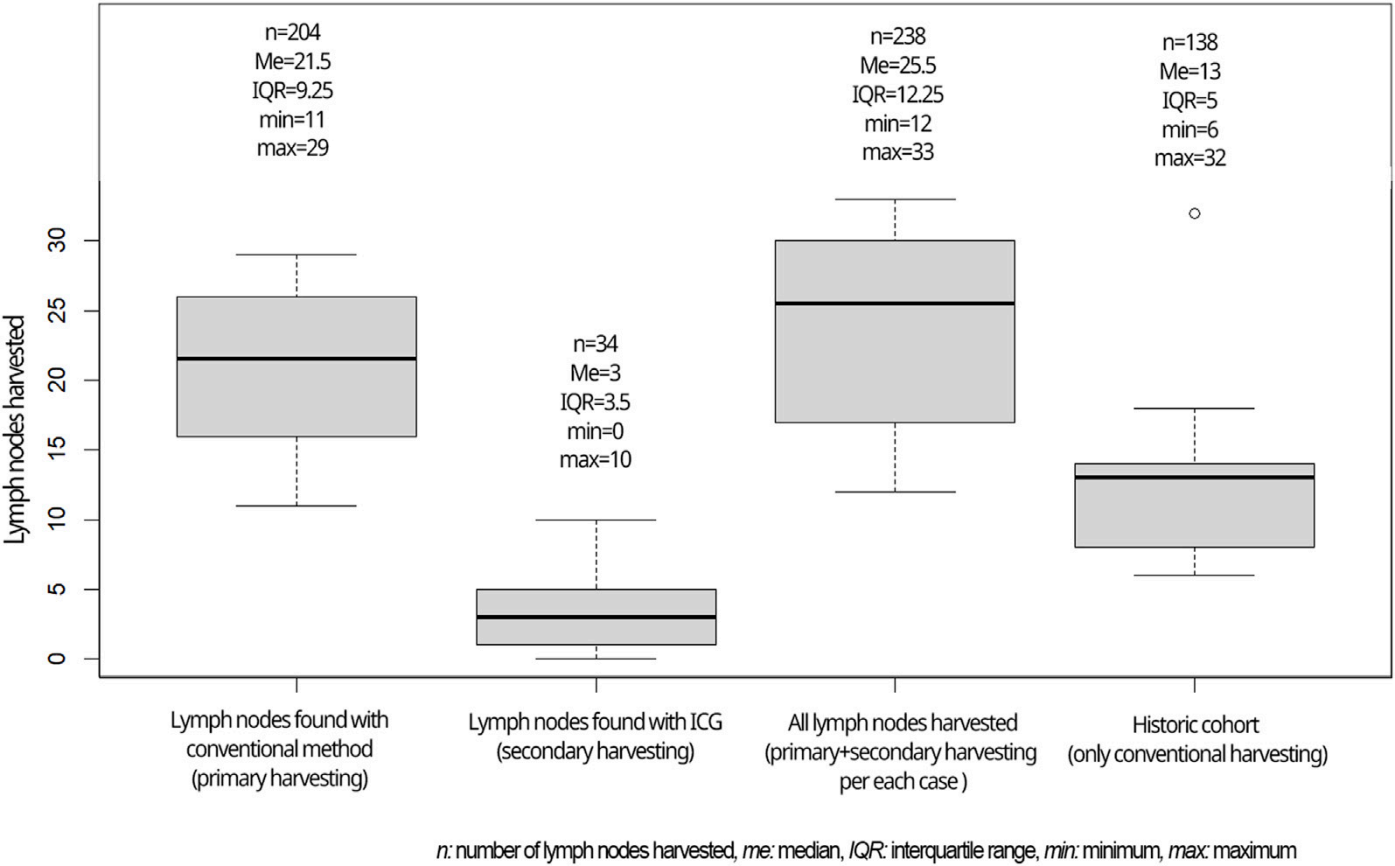
Lymph nodes harvested in the current study compared to the historical cohort.

After propensity score matching, the median lymph node yield was significantly higher in the study group even without ICG assistance than in the control group of a historical cohort (Figure 6) (median of 21.5 vs. 13, *p* = 0.052, U = 24, r = 0.6). In total, 30% of the cases in the historical cohort had suboptimal nodal staging (less than 12 lymph nodes assessed), while only 2 cases (20%) had inaccurate nodal staging in the traditional work-up during the study period, both of which were improved above 12 nodes with ICG supplementation.

In our current study, the accuracy was 80%, in the propensity-matched historical cohort it was 70%.

### Adverse events

Because ICG-assisted lymph node dissection was performed *ex vivo*, no adverse events were recorded regarding the assessments. Given that a single vial of ICG is sufficient for intraoperative perfusion assessment and *ex vivo* lymph node identification, the technique has no additional cost.

## Discussion

Indocyanine green as a fluorescent dye is commonly used in medical imaging and diagnostic procedures. It absorbs light in the near-infrared range (wavelengths of 700–900 nm), which is beyond the visible spectrum. Near-infrared light is less scattered and absorbed by tissues compared to visible light, resulting in deeper tissue penetration. The present study aimed to evaluate the feasibility and performance of *ex vivo* intraarterial ICG perfusion-based fluorescence lymph node identification in colorectal cancer.

The number of examined lymph nodes is highly prognostic [13] in addition to being a strong predictive factor affecting the postoperative multidisciplinary team decision regarding oncotherapy [14]. In cases of inaccurate N0 staging (examined nodes less than 12), patients may fail to receive the beneficial adjuvant therapy (which is routine in Hungarian oncological practice) or may be the target of unnecessary overtreatment (in some Western-European countries) [15].

In our pilot study of 10 consecutive cases of colorectal resection, ICG-FI significantly improved the number of retrieved lymph nodes, however the number of positive (metastatic) lymph nodes was not affected. Considering that the minimum lymph node yield for accurate staging according to the AJCC Cancer Staging System is at least 12, our data show that even without the help of ICG, this requirement is fulfilled in the majority of the current study. This makes the beneficial effect of ICG-FI even more impressive.

Our results show a significantly higher lymph node yield when compared to our retrospectively analyzed past results of conventional (manual) tissue harvesting. Therefore we have proved that the high success rate of lymph node retrieval within the study is at least partly due to a Hawthorne effect.

Consequently, the use of the ICG FI technique may be beneficial due to the higher number of identified lymph nodes, which in turn will result in more precise staging where accurate lymph node staging is more difficult to achieve on a regular basis.

Our results support the finding of other researchers that a significant proportion (up to 50%) of metastatic lymph nodes are smaller than or equal to 5 mm in diameter [16]. Therefore, the easy-to-perform ICG-FI is well expected to increase the positive lymph node yield.

Accuracy in pathological staging is defined as at least 12 non-metastatic lymph nodes found or any number of lymph nodes but with N1-2 stage [6, 17]. This is the primary requirement for the appropriate decision on the need for adjuvant chemotherapy, therefore it can also have a long-term oncological impact [18–20].

Although ICG-FI requires special equipment (laser, camera, dye), the same setup can be used for a variety of tasks. There is a good possibility that it will be used routinely both in the operating room and for pathological examinations in the future.

## Conclusion

ICG fluorescence can improve the accuracy of lymph node staging. It identified additional lymph nodes that were not detected by traditional pathological examination, indicating that this technique has the potential to help optimize treatment planning and improve patient outcomes.

Centers with a lower number of routinely retrieved lymph nodes after colorectal resection, in addition to cases following neoadjuvant chemo-irradiation may benefit most from this technique.

Larger studies are needed to validate these findings and to determine the optimal protocol for using ICG fluorescence to improve histologic specimen processing after colorectal surgery.

Comparison of ICG-FI with other techniques for the same purpose (methylene blue intra-arterial injection, fat clearance techniques, use of pathology assistants, etc.) will be a task for the future.

## Data Availability

The raw data supporting the conclusions of this article will be made available by the authors, without undue reservation.
